# Pre-existing neutralizing antibody mitigates B cell dysregulation and enhances the Env-specific antibody response in SHIV-infected rhesus macaques

**DOI:** 10.1371/journal.pone.0172524

**Published:** 2017-02-21

**Authors:** Juan Pablo Jaworski, Peter Bryk, Zachary Brower, Bo Zheng, Ann J. Hessell, Alexander F. Rosenberg, Tong Tong Wu, Ignacio Sanz, Michael C. Keefer, Nancy L. Haigwood, James J. Kobie

**Affiliations:** 1 Oregon National Primate Research Center, Oregon Health & Science University, Beaverton, Oregon, United States of America; 2 Department of Microbiology and Immunology, University of Rochester Medical Center, Rochester, New York, United States of America; 3 Division of Infectious Diseases, Department of Medicine, University of Rochester Medical Center, Rochester, New York, United States of America; 4 Divsion of Allergy, Immunology & Rheumatology, Department of Medicine, University of Rochester Medical Center, Rochester, New York, United States of America; 5 Department of Biostatistics and Computational Biology, University of Rochester Medical Center, Rochester, New York, United States of America; 6 Lowance Center for Human Immunology and Division of Rheumatology, Department of Medicine, Emory University, Atlanta, Georgia, United States of America; University of Pittsburgh Centre for Vaccine Research, UNITED STATES

## Abstract

Our central hypothesis is that protection against HIV infection will be powerfully influenced by the magnitude and quality of the B cell response. Although sterilizing immunity, mediated by pre-formed abundant and potent antibodies is the ultimate goal for B cell-targeted HIV vaccine strategies, scenarios that fall short of this may still confer beneficial defenses against viremia and disease progression. We evaluated the impact of sub-sterilizing pre-existing neutralizing antibody on the B cell response to SHIV infection. Adult male rhesus macaques received passive transfer of a sub-sterilizing amount of polyclonal neutralizing immunoglobulin (Ig) purified from previously infected animals (SHIVIG) or control Ig prior to intra-rectal challenge with SHIV_SF162P4_ and extensive longitudinal sampling was performed. SHIVIG treated animals exhibited significantly reduced viral load and increased *de novo* Env-specific plasma antibody. Dysregulation of the B cell profile was grossly apparent soon after infection in untreated animals; exemplified by a ≈50% decrease in total B cells in the blood evident 2–3 weeks post-infection which was not apparent in SHIVIG treated animals. IgD+CD5+CD21+ B cells phenotypically similar to marginal zone-like B cells were highly sensitive to SHIV infection, becoming significantly decreased as early as 3 days post-infection in control animals, while being maintained in SHIVIG treated animals, and were highly correlated with the induction of Env-specific plasma antibody. These results suggest that B cell dysregulation during the early stages of infection likely contributes to suboptimal Env-specific B cell and antibody responses, and strategies that limit this dysregulation may enhance the host’s ability to eliminate HIV.

## Introduction

One of the goals of vaccination is to establish B cell memory that can be efficiently recruited upon virus exposure to develop antibodies that are directed at conserved epitopes in order to prevent or control infection, and this goal has been a substantial hurdle for the human immunodeficiency type 1 (HIV-1) vaccine field. The only human vaccine trial to date that has shown protective efficacy, modest at 31%, is RV144 in Thailand where a reduction in infection risk was correlated with the presence of anti-V1 and–V2 antibodies [1], and only a low level of neutralizing antibodies (NAbs) were observed [2]. While several studies in animal models have shown evidence confirming the role of NAbs in protection and control of HIV-1, no experimental vaccine has achieved the goal of inducing a humoral response that could be expected to protect humans against the global diversity of infecting isolates. Passively transferred human polyclonal or monoclonal NAbs (NmAbs) have been widely used to test for protection against infection in nonhuman primates (NHP) in simian-human immunodeficiency virus (SHIV) models of HIV-1 infection. In those settings, passive administration of NmAbs was able to fully protect against intravenous [3] or mucosal [4–9] SHIV challenge. Furthermore, there is evidence that potent NmAbs can lower viremia in chronic infections in NHP models [10, 11] and humans [12, 13]. Notably, we have recently shown that a combination of potent NmAbs administered 24 h after viral exposure can intercept replicating viral foci, prevent the establishment of a permanent reservoir, and mediate the clearance of the virus from the host within 14 days [6]. A confirmatory study later reported similar findings using pre-exposure with NmAb [14], and both studies are exemplary in the demonstration of the dual functionality of antibodies in the setting of HIV-1 infection, as the killing of infected cells was likely accomplished by Fc-mediated effector functions.

During natural HIV-1 infection the antibody response is delayed and NAbs only appear after 12 weeks of infection [15]. In addition to some of the most effective evasion mechanisms described to date, including: (i) expression of a limited number of functional Env on the surface of the virion, (ii) remarkable diversity, (iii) glycosylation shield and (iv) conformational flexibility, HIV-1 [16, 17] and SIV [18, 19] have been shown to cause acute damage to the B cells in peripheral blood and in the gut. The B cell dysregulation observed in these studies was characterized by polyclonal activation, terminal differentiation and apoptosis. As a consequence of acute B cell dysfunction the host humoral response to HIV and other pathogens might be affected [20].

Although sterilizing immunity mediated by pre-formed abundant and potent antibodies is the ultimate goal for B cell-targeted HIV vaccine strategies, scenarios that fall short of this may still confer beneficial immunity, and it is possible that HIV-1 vaccines may only achieve sub-sterilizing humoral immunity upon exposure. This circumstance could be the consequence of insufficient quantities of antibody being present due to limited persistence, inefficient induction of the most effective specificities and potency, or limited affinity as a result of poor cross-reactivity between the vaccine strain and infecting strain. Following HIV transmission, there is a limited window of opportunity for the adaptive immune response to potentially prevent the development of chronic infection, making it imperative to understand the dynamics of the B cell response during the early stages of infection and define mechanisms of enhancing its activity.

New SHIV models are needed to investigate whether passively transferred antibodies or antibodies elicited by vaccination that fall short of providing sterilizing immunity influence the endogenous immune response and its ability to control infection. We have utilized the model developed by Haigwood et al., whereby a sub-sterilizing dose of polyclonal neutralizing IgG isolated from a previously infected animal is passively transferred prior to SIV [21] or SHIV [22, 23] mucosal challenge. In this setting, passive transfer of neutralizing IgG treatment (SHIVIG) reduced both plasma and peripheral blood mononuclear cell (PBMC)-associated viremia and preserved CD4^+^ T cells in infant rhesus and pigtailed macaques challenged with SHIV_SF162P3_ [22, 23]. Interestingly, SHIVIG treatment in newborn rhesus not only reduced the amount of integrated virus in peripheral blood cells, but also preserved B cells, resulting in accelerated and stronger NAb and antibody-dependent cell-mediated viral inhibition (ACDVI) development and dramatically guarded the infants against rapid disease progression and death [23].

The dynamics of this model in adult rhesus macaques and the influences on the B cell response remained undetermined. We hypothesize that protection against HIV-1 infection will be powerfully influenced by the magnitude and quality of a rapid B cell response. Here, we have investigated the impact of NAb pre-exposure treatment on early SHIV_SF162P4_ infection in adult macaques and its protection of the B cell compartment.

## Materials and methods

### Ethics statement

All animal work was conducted in accordance with the recommendations of the Weatherall report, "The use of non-human primates in research." Specifically, the research is regulated by the Office of Laboratory Animal Welfare and the United States Department of Agriculture and the National Institutes of Health Guide for Care of Laboratory Animals, and the facilities at the Oregon National Primate Research Center are accredited by the American Association for Accreditation of Laboratory Animal Care. There is no alternative to the use of nonhuman primates to study the pathogenesis of viruses related to HIV-1 and how the virus stimulates the immune responses *in vivo*. The experimental protocols were approved by the Oregon Health & Science University Institutional Animal Care and Use Committee. All macaques were pair-housed for this study and provided with enrichment activities and species appropriate treats. All procedures involving potential pain were performed with the appropriate anesthetic or analgesic. The number of animals used in this study was scientifically justified based on statistical analyses of virological and immunological outcomes.

### Macaques

Adult male *Macaca mulatta* (rhesus macaques) were obtained from the breeding colony and raised at the Oregon National Primate Research Center in Beaverton, Oregon, U.S.A. (ONPRC). Baseline peripheral blood was sampled 1 week before infection and either 1 day before (NIgG group) or immediately before (SHIVIG group) infection, and then at regular intervals throughout the study. For IgG administration and blood draws, animals were anesthetized with 8–20 mg/kg ketamine administered intramuscularly. Animals were monitored daily for health, appetite and species-normal behavior. Weight and lymph node observations were performed weekly. None of the animals developed clinical signs during the study period. All macaques infected with lentiviruses were euthanized at the conclusion of the study, as this is an infectious disease and animals must be cared for in biosafety level 2-plus at all times. For euthanasia, macaques are anesthetized, then administered >50 mg/kg sodium pentobarbital administered intravenously. Following this, animals are exsanguinated via the distal aorta. This method is consistent with the recommendations of the American Veterinary Medical Association Guidelines for Euthanasia.

### IgG preparations

IgG purification was performed as previously described [24]. Normal IgG (NIgG) was purified from 1 liter (L) of pooled plasma from simian immunodeficiency virus (SIV) negative adult rhesus macaques screened for the absence of reactivity to HIV-1 SF162 gp140 (by ELISA) and absence of neutralization against HIV_SF162_ pseudovirus (TZM-bl assay). SHIVIG formulation was purified from 1 L of pooled plasma obtained from terminal bleeds of four rhesus macaques that were infected with SHIV_SF162P4_ for 1 year and that developed neutralizing antibodies against the infecting SHIV. SHIVIG neutralization activity was measured against HIV_SF162_ pseudovirus in the TZM-bl assay and a 50% inhibitory concentration of 0.32 μg/ml was obtained (compared to 0.015 of the reference NmAb b12). Purity was >90% as determined by SDS-PAGE.

### Virus challenge and IgG administration

In this study, we used SHIV_SF162P4_ (passage 4) virus, which has been described elsewhere [25, 26]. We obtained SHIV_SF162P4_ through the NIH AIDS Research and Reference Reagent Program, Division of AIDS, National Institute of Allergy and Infectious Diseases, NIH (catalog “SHIV_SF162P4_ NIH 2” (2006); contributors J. Harouse, C. Cheng-Mayer and R. Pal). Ten adult male rhesus macaques were divided into two groups of 5 animals per group. Purified IgG was delivered subcutaneously at multiple sites around the scruff of the neck and the back of the animals 24 hours before virus inoculations. Normal IgG was given at a dose of 150 mg/kg and SHIVIG was dosed at 25 mg/kg. SHIVIG dose was based on the potency of neutralization against HIV-1 SF162 (TZM-bl assay) and was chosen to provide less than sterilizing immunity, leaving animals at risk of infection. Following IgG administration intrarectal virus exposures were delivered in two inoculations 24 h apart. Virus was inoculated at 100% animal infectious doses (AID_100_). AID was determined in a titration experiment performed by Dr. Cheng-Mayer (personal communication. Animals were monitored for 16 weeks for clinical signs of disease, including lymph node palpation and measurement, weight, appetite, etc. Blood samples were taken at weekly, bimonthly, or monthly intervals to determine lymphocyte subsets, antibody responses, and plasma viral load.

### Plasma viral load

Viral stock RNA and plasma viral RNA samples were extracted using the QiaAmp viral RNA extraction kit (Qiagen) per manufacturer’s instructions. RNA copy number was measured by quantitative RT-PCR (RT-qPCR). RT reactions consisted of 500 μM dNTPs, 2.5 ng/μl random hexamers, 0.6 U/μl RnaseOut, 5.7 U/μl SuperScript III (Invitrogen) in a total volume of 14 μl. RT reaction conditions included 25°C for 10 min, followed by 42°C for 50 min, then 85°C for 5 min. To quantify cDNA, 2 μl of the RT reaction was used in a quantitative PCR (total 30 μl). Briefly, The reaction contained TaqMan universal PCR master mix (ABI, Norwalk, CT), 500 nM forward and reverse primers (GAG5f, 5´-ACTTTCGGTCTTAGCTCCATTAGTG-3´; GAG3r, 5´-TTTTGCTTCCTCAGTGTGTTTCA-3´), and 200 nM TaqMan probe labeled with a 5´ 6-carboxyfluorescein fluorescent reporter dye and a 3´ quencher (5´-FAM-TTCTCTTCTGCGTGAATGCACCAGATGA-TAMRA-3´). Real-time PCR was performed on an ABI 7500 machine (ABI) with the following cycling conditions: 2 min at 50°C, 95°C for 10 min, and then 45 cycles at 95°C for 15 s and at 60°C for 1 min. Standard for RNA was an *in vitro* transcript of plasmid p239gag containing *Kpn*I-*BamH*I SIV gag fragment from SIVmac239 (gift of J. Lifson and M. Piatak). Ten-fold dilutions of this standard were made from 1 x 10^6^ copies μl^-1^ to 10 copies μl^-1^. A final 2-fold dilution was made to obtain 5 copies μl^-1^. High, intermediate and weak positive controls, as well as negative controls, were included in each plate.

### Pseudovirus construction

HIV_SF162_ *Env* clone contained within the pEMC* expression plasmid was co-transfected with the *Env*-deleted viral backbone plasmid Q23Δ*Env* (kindly provided by Dr. Julie Overbaugh [27]) in 293T cells [23].

### TZM-bl neutralization assay

Plasma samples from each animal were tested at all available time-points for neutralizing activity using the 96-well TZM-bl neutralization assay described previously [28].

### ELISA

Enzyme-linked immunosorbent assay (ELISA) was used to assess the presence of gp140-specific IgG antibodies as previously described [29].

### Flow cytometry

For global B cell phenotypic analysis, peripheral blood mononuclear cells (PBMCs) were stained with anti-CD19-APC-AlexaFluor700 (J3-119, Beckman Coulter, Brea, CA), anti-CD20-APC-Cy7 (L27, BD Biosciences, San Jose, CA), anti-CD4-Qdot605 (S3.5, Invitrogen, Carlsbad, CA), anti-IgD-FITC (Dako, Carpinteria, CA), anti-IgG-biotin (Jackson Immuno, West Grove, PA), anti-CD38-APC (OKT10, NHP Reagent Repository), anti-CD27-Qdot655 (CLB-27/1, Invitrogen), anti-CD10-Qdot800 (MEM-78, Invitrogen), anti-CD95-Pacific Blue (DX2, Biolegend, San Diego, CA), anti-CD21-PE-Cy5 (B-ly4, BD Biosciences), anti-CD24-PE-AlexaFlour610 (SN3, Invitrogen), anti-CD5-PE (CD5-5D7, Invitrogen), anti-PD1-PerCP-eAlex710 (eBioJ105, eBioscience, San Diego, CA), anti-CD8-Qdot705 (3B5, Invitrogen), anti-CD11c-PE-Cy7 (3.9, eBioscience), anti-CD14-PE-Cy5.5 (61D3, Abcam, Cambridge, UK), and Live/Dead Yellow (Invitrogen). One-to-five million total events per sample were collected on an LSRII instrument (BD Biosciences) and analysis performed using FlowJo software (Treestar, Inc, Ashland, OR). Total PBMC were gated on lymphocytes using FSC and SSC. Live/Dead stain and anti- CD4, CD8, and CD14 were used to exclude dead cells and non-B cells, respectively. B cells were identified by the expression of CD19 and CD20, and B cell subset analysis performed using remaining markers by sequential manual gating applied to all samples in a consistent manner.

### Statistical analysis

Two-tailed t test was used to compare groups at each time point, assuming normality. To analyze the kinetics of the B cell subsets as determined by flow cytometry, functional data analysis techniques were used. Functional principal component analysis was performed on both the un-smoothed and B-spline smoothed versions of all variables, including 128 B cell subsets and their percentages changed from baseline, and plasma Env-specific binding antibody response, for the two treatment groups separately. The resultant functional eigenvalues of the B cells were correlated with the resultant functional eigenvalues of the plasma Env-specific binding antibody outcome for each treatment group. The Env-specific binding antibody outcome eigenvalue was determined with the exclusion of week 0 through week 5 values to eliminate the direct contribution of passively transferred antibody to the kinetic profile. The correlation coefficients of the two groups were compared with a two-tailed z test. Additionally, the kinetics of B cells and the plasma Env-specific binding antibody response of the two treatment groups are compared using a permutation t test with 200 random permutations. Statistical analyses were performed using Prism 6.0 software (GraphPad Software, La Jolla, CA) and R 3.3.1 software (R Core Team (2016), URL https://www.R-project.org/) and significance was taken as p<0.05.

## Results and discussion

### Pre-existing neutralizing antibody limits viral burden and acute CD4 expansion

**Fig 1:**
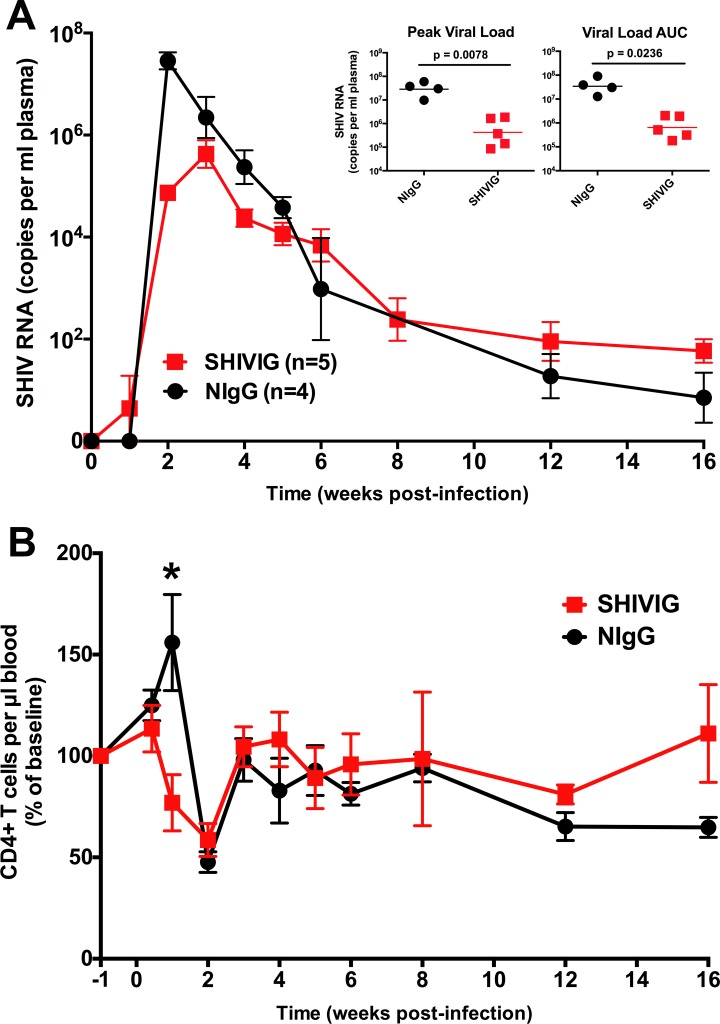
Effect of passively transferred neutralizing IgG on plasma viral load and CD4^+^ T cells in SHIV_SF162P4_ infected macaques. Male rhesus macaques were treated with NIgG (n = 4) or SHIVIG (n = 5) and blood samples collected at regular intervals after viral exposure. (**A**) RNA was isolated from plasma, and viral SHIV RNA was quantified by RT-PCR. (**B**) CD4^+^ T cell count was determined by flow cytometry and normalized to percentage of baseline value. Baseline value was defined as the average value of the -1 w.p.i and either -1 day p.i. (NIgG group) or 0 day p.i. (SHIVIG group). Symbols represent group mean±SEM. * indicates significant difference (p<0.05) between groups at indicated time point as determined by two-tailed t-test.

Male rhesus macaques received normal IgG (n = 5) or IgG isolated from SHIV_SF162P4_ infected animals (n = 5) that had developed neutralizing antibodies, and which was delivered at a sub-sterilizing dose, and are henceforth referred to as NIgG and SHIVIG. Previous studies indicate that the majority of SHIVIG is decayed by 6 weeks after administration [21, 23, 30]. The animals were intra-rectally challenged with high dose SHIV_SF162P4_ and monitored for plasma viremia (**Fig 1A**). One of the NIgG treated animals did not develop viremia and was therefore excluded from the study. Peak viral load in the NIgG group (n = 4) occurred at 2 weeks post infection (w.p.i.), but in the SHIVIG group (n = 5) viremia was delayed to 3 w.p.i. and was significantly lower (3.4x10^7^ vs. 8.2x10^5^ RNA copies/ml, p = 0.0078). A reduction in total plasma viral burden, measured as area under the curve (AUC) during the 16 weeks of study, was also significantly lower in the SHIVIG group (4.3x10^7^ vs. 9.9x10^6^, p = 0.0236). Moreover, when compared, peripheral blood CD4^+^ T cells significantly expanded (p = 0.0317) in the NIgG group during the first week following challenge, but not in the SHIVIG group (**Fig 1B**). This blunted CD4^+^ T cell expansion in SHIVIG treated animals may have contributed to the reduced viral burden in the SHIVIG group, through potentially limiting the abundance of CD4+ T cells available to be infected. Following peak viremia, the NIgG group exhibited a progressive and significant reduction in CD4^+^ T cells (~35% of baseline, p = 0.006 at wk 16). In contrast, the CD4^+^ T cells remained relatively stable in the SHIVIG group, despite comparable levels of viremia from 6 w.p.i on. These results suggest the presence of SHIVIG during acute infection confers a sustained impact on the CD4^+^ T cell dynamics, even as the antibody decays. By reducing initial viral burden, SHIVIG may blunt the amplification loop of increased immune activation that results in additional activated CD4^+^ T cells as virus targets.

### Pre-existing neutralizing antibody prevents B cell dysregulation

**Fig 2:**
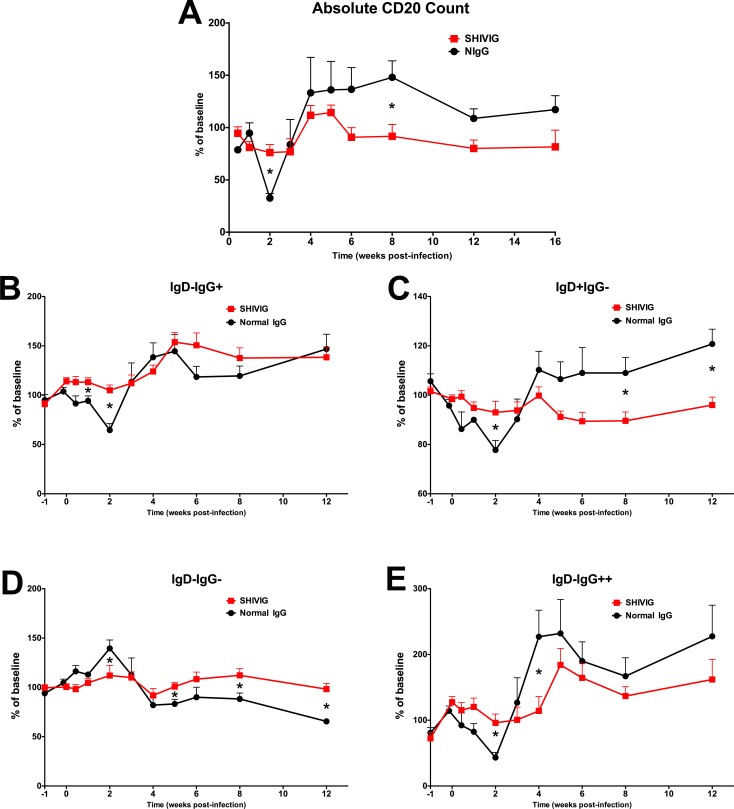
SHIVIG treatment mitigates gross B cell dysregulation. The absolute peripheral blood total CD20+ B cell count (**A**) and frequency of CD19+CD20+ IgD/IgG B cell subsets (**B-E**) was determined by flow cytometry. Symbols represent group mean+SEM. * indicates significant difference (p<0.05) between groups at indicated time point as determined by two-tailed t-test.

HIV-1 and SIV infection are known to dramatically alter the phenotype and function of B cells [16–19], however it is unclear how B cell dysregulation during acute infection impacts the development of Env-specific antibody. Total peripheral blood B cells were analyzed longitudinally to assess the impact of infection and SHIVIG on their dynamics, specifically monitoring changes from baseline. Each animal’s baseline is the average value of their two pre-infection samples. The absolute CD20^+^ total B cell count revealed a significant decrease in both the NIgG group (p = 0.0006) and SHIVIG (p = 0.0355) group at 2 w.p.i compared to baseline, that remained decreased in the SHIVIG groups at 3 w.p.i., corresponding to peak viral load. The NIgG group exhibited a more severe and significant decrease in CD20+ total B cells at 2 w.p.i. compared to the SHIVIG group (~67% vs. ~24%, p = 0.003) (**Fig 2A**). This acute decrease in total B cells was followed in the NIgG group by a progressive increase in total B cells sustaining a ~20%– 35% increase over baseline level through 16 w.p.i., and was significantly greater (p = 0.020) at 8 w.p.i. compared to the relatively stable B cell totals at subsequent timepoints in the SHIVIG group. These results indicate that SHIVIG stabilized the frequency of total B cells during infection and are consistent with our previous findings in other models [22, 23].

To more finely assess the dynamics of the B cell compartment, the frequency of B cell subsets defined by IgD and IgG expression were monitored and are described as follows: (i) IgD+IgG- (IgD+), which primarily contains naïve B cells and IgM memory B cell subsets, (ii) IgD-IgG+ (IgG+), the predominantly switched memory B cell compartment, (iii) IgD-IgG- which primarily contains IgA memory B cells [31–34], and (iv) IgD-IgG++ a minor population, which may represent an activated memory-like subset. Utilizing even this somewhat limited set of phenotypic markers, significant dysregulation within the B cell compartment was observed (**Fig 2**). In NIgG treated animals, a progressive decrease in IgD+, IgG+, and IgD-IgG++ populations was measured during the early stage of infection and was most severe at 2 w.p.i. corresponding with peak viral load (**Fig 2B, 2C and 2E**). In contrast, these populations remained relatively stable in the SHIVIG treated animals during the first 4 w.p.i, as indicated by significant differences between groups at multiple time points. During the later stage of infection, the frequency of IgG+ (**Fig 2B**) and IgD-IgG++ (**Fig 2E**) populations surpassed baseline levels in both groups, and the frequency of the IgD+ population in NIgG treated animals surpassed baseline level and was significantly higher compared to the SHIVIG treated animals at 8 and 12 w.p.i. (**Fig 2C**). Although the IgD-IgG- population remained relatively stable in the SHIVIG group in contrast to the other populations, an increase during the acute stage and progressive decline in the late stage was observed in the NIgG group (**Fig 2D**). These results suggest that SHIVIG mediated reduction in acute viremia is preventing global B cell dysregulation.

### High-resolution B cell phenotyping

**Fig 3:**
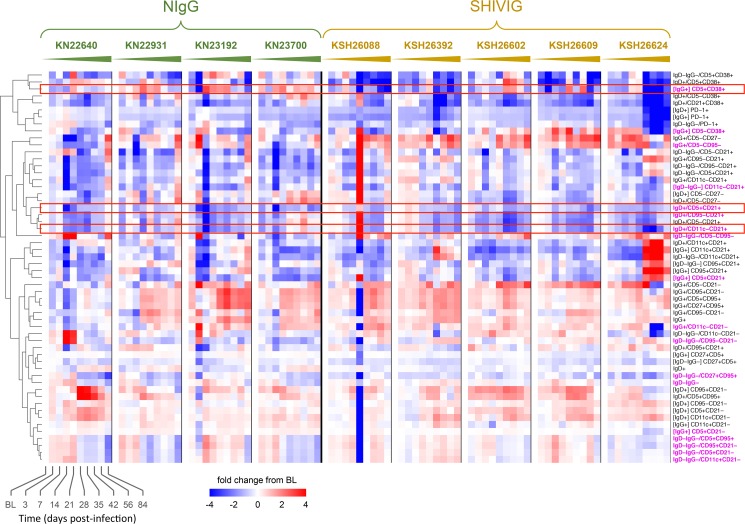
High-resolution longitudinal B cell phenotypic profile. Heatmap shows data corresponding to the change (log2) from baseline value for each subset frequency at each time point. Paired two tailed t-test for all post-baseline time points vs. baseline was computed independently for NIgG and SHIVIG groups; populations shown had at least one significant time point p<0.01 compared to baseline. Subsets were clustered hierarchically based on Euclidean distance and complete linkage. Magenta subset label indicates a significant difference of at least one time point for the log2 change from baseline between NIgG and SHIVIG groups at p<0.01 as determined by two-tailed unpaired t-test. [X] indicates subset is measured as the frequency of X parent population, otherwise parent population is total CD19+CD20+ B cells. Rectangle outline is used to emphasize select subsets.

Using a 17-color flow cytometry panel we evaluated the extended phenotype of the peripheral blood B cells longitudinally, focusing on subsets whose frequency was substantially changed from baseline (p<0.01) in at least one group and at least one time point after infection (**Fig 3**). As soon as 3 days p.i. the frequency of several B cell subsets differed between groups, most evident was the IgD+CD5+CD21+ (p = 0.007), which comprised ~5% of the total B cell compartment at baseline, and decreased by ~50% in NIgG animals, but remained relatively stable in SHIVIG animals. The IgD+CD5+CD21+ subset continued to decline in NIgG animals and at 14 days p.i. was at ~30% of baseline, remaining significantly lower than in SHIVIG animals (p = 0.009), but returned to near baseline by 28 days p.i. At 21 days p.i. the IgD+CD11c-CD21+ subset, which comprised ~15% of the total B cell compartment at baseline decreased by ~50% in NIgG animals, which was significantly lower than in SHIVIG animals (p = 0.005). In contrast at this time point the IgG+CD5+CD38+ subset, which comprised ~1% of the total B cell compartment at baseline had increased by ~200% in NIgG animals, and returned to near baseline by 56 days p.i. while remaining stable in SHIVIG animals (p = 0.0006), with a gradual overall decline during the 12 week period.

### SHIVIG treatment results in higher titers of de novo plasma binding antibody

**Fig 4:**
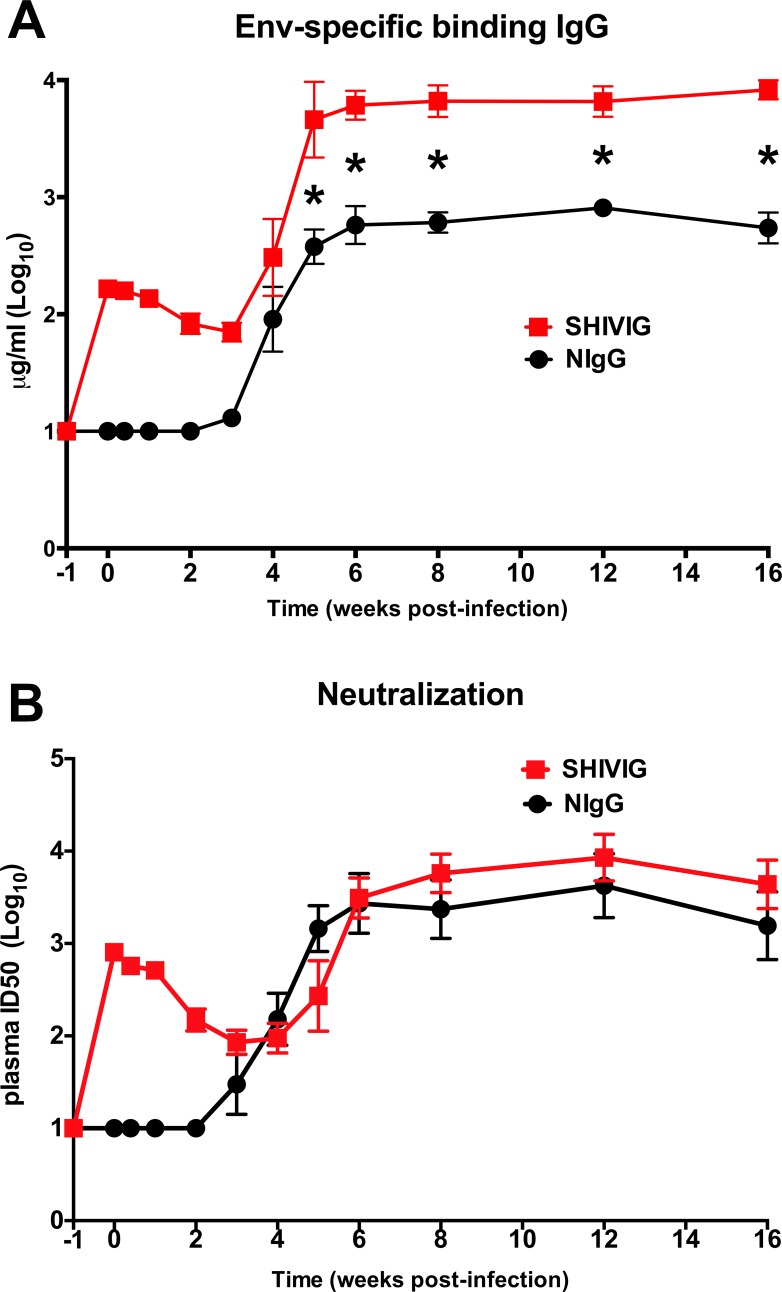
SHIVIG treatment enhances plasma HIV-1 SF162 envelope binding antibody response. (**A**) Binding antibody (BAb) titer was determined by IgG ELISA against HIV-1 SF162 gp140 envelope protein. (**B**) Neutralization antibody (NAb) titer against HIV_SF162_ pseudovirus was determined by TZM-bl neutralization assay. Symbols represent group mean±SEM. * indicates significant difference (p<0.05) between groups at indicated time point as determined by two-tailed t-test.

Consistent with our previous findings with passive transfer prior to SHIV in infant macaques [22, 23], we observed increased Env-specific binding plasma IgG antibody (BAb) in SHIVIG treated animals (**Fig 4A**). The SHIVIG group had significant (p<0.05) and ~10-fold higher BAb titer through week 16 compared to NIgG. However, in contrast to our previous findings in infants, SHIVIG had minimal impact on plasma neutralizing antibody titers (**Fig 4B**) in this study. The overall reduced viral burden in the SHIV_SF162P4_ infection model of adult rhesus macaques contrasts with the high and sustained viremia observed in infant macaques infected with SHIV_SF162P3_ or adult macaques infected with SIV, and may have masked the full potential of SHIVIG to enhance the B cell response.

### Kinetics-based analysis identifies B cell correlates of plasma binding antibody

**Fig 5:**
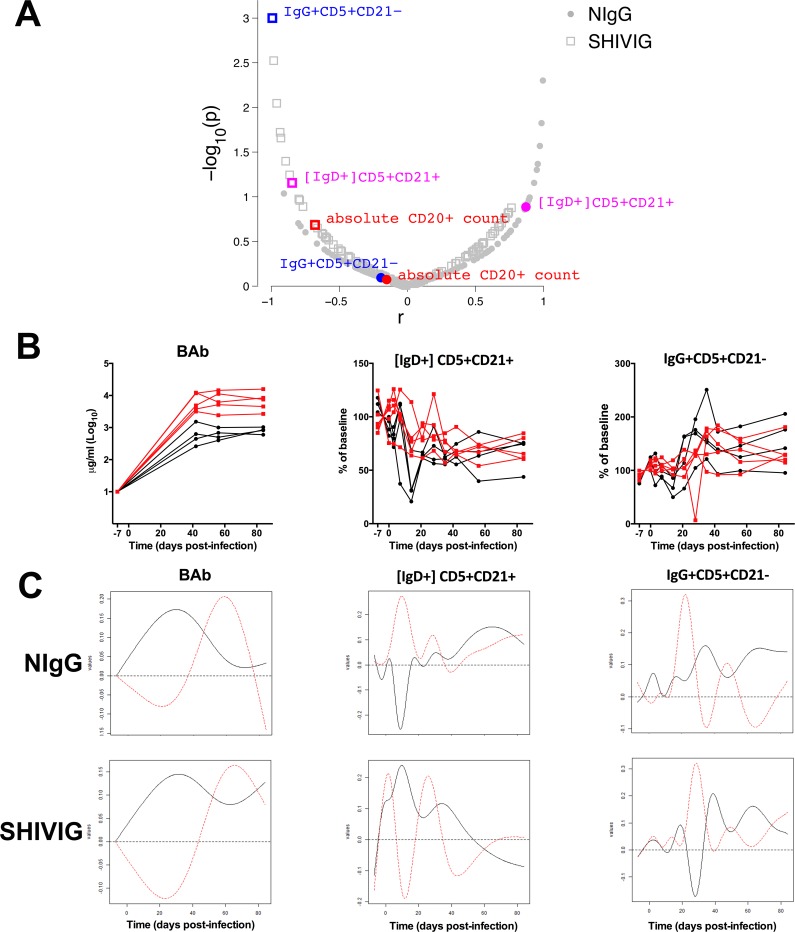
Kinetic analysis of binding antibody association with peripheral B cell subsets. **(A)** Correlation of the eigenvalues of the kinetic profile of the B cell subsets (n-128) and plasma Env-specific binding Ab (BAb). Red indicates significant overall (group independent) correlation (p<0.05) of B cell subset and BAb eigenvalues. Blue indicates significant difference (p<0.05) between groups and their correlation with BAb. Magenta indicates both significant overall correlation with BAb and significant difference between groups. **(B)** Individual kinetic profile of *de novo* BAb, and B cell subsets. Each line represents an individual animal. Black indicates NIgG, red indicates SHIVIG. **(C)** Eigenvalues of BAb and B cell subsets.

**Fig 6:**
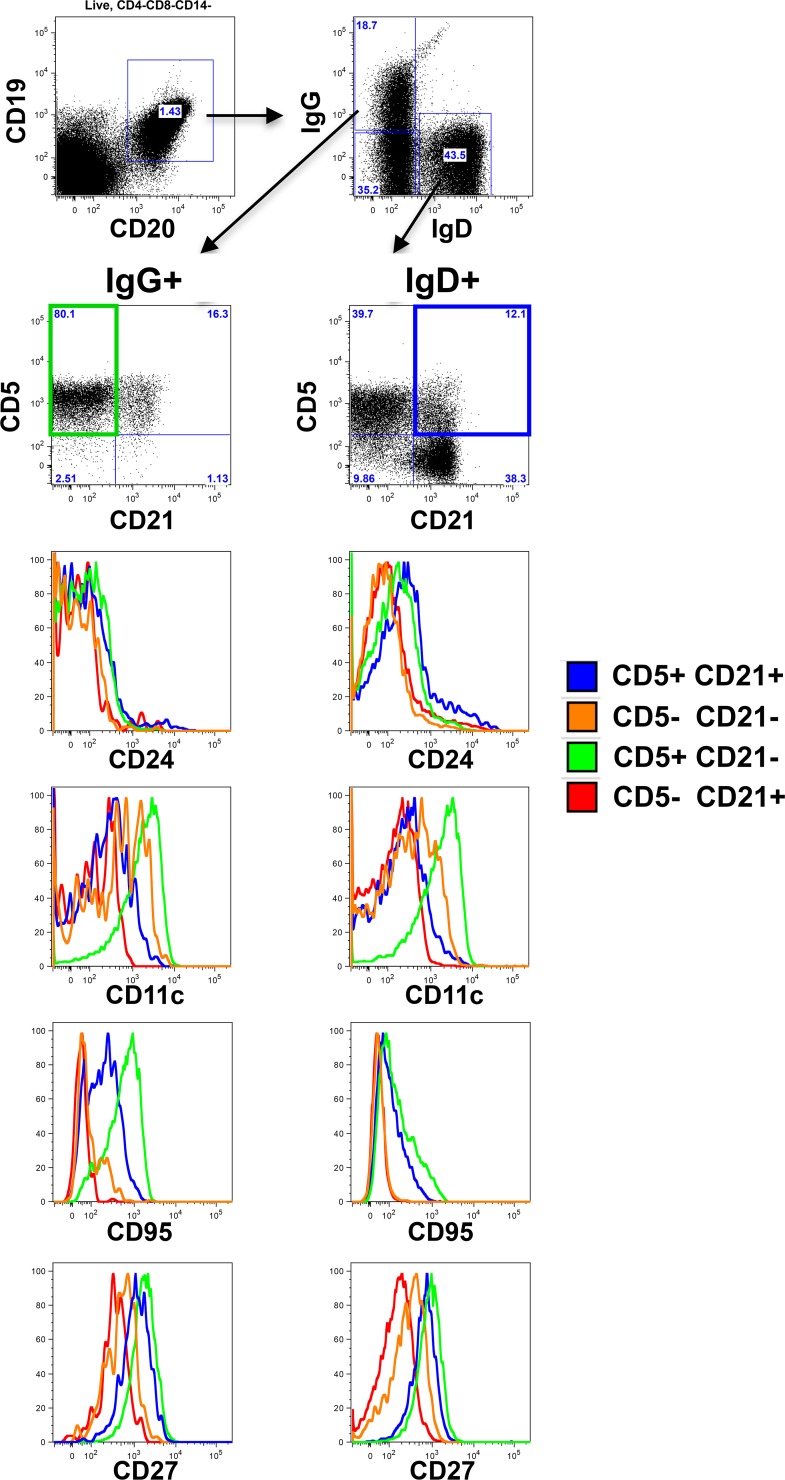
Detailed phenotype of CD21^+^ and CD5^+^ B cell subsets. B cell flow cytometry gating strategy and marker expression profile of a representative baseline PBMC sample.

To distill peripheral B cell subsets whose kinetics after infection are most closely associated with the kinetics of Env-specific plasma antibody development, we utilized functional principal components analysis. A functional eigenvalue was obtained for the kinetic profile of each B cell subset, which was then correlated with the functional eigenvalue of the BAb kinetic profile. When assessing all animals together, ignoring their group association, only the kinetic profiles of absolute CD20^+^ B cell count (r = 0.797, p = 0.01) and IgD+CD5+CD21+ (r = 0.843, p = 0.004) significantly correlated with BAb (not shown). When comparing the two groups for their individual relationship between the kinetic profiles of B cell subset and BAb, only IgD+CD5+CD21+ (p = 0.035) and IgG+CD5+CD21- (p = 0.034) were significantly different between groups (**Fig 5A**). IgD+CD5+CD21+ kinetics (black solid curve decreases in early stage and then increases) correlated with BAb kinetics (increases in early state and decreases later on) in NIgG and also correlated with BAb kinetics although in an opposite manner (black curve increases in early stage and then decreases) in SHIVIG, with a significant group difference (p = 0.035) (**Fig 5C**). The distinct kinetics of the IgD+CD5+CD21+ B cell subset between groups are also apparent when observing their change in frequency from baseline in the individual animals (**Fig 5B**). Closer phenotypic analysis of these subsets (**Fig 6**) revealed the IgG+CD5+CD21- B cell subset, which increases in the peripheral blood during the late stages of infection in the NIgG group, has increased CD95, CD27, and CD11c expression relative to other IgG+ subsets, suggesting an activated IgG+ memory subset.

The IgD+CD5+CD21+ subset has increased expression CD95, CD27, and CD24 relative to other IgD+ subsets, and suggests it may be an activated naïve or marginal zone (MZ)-like subset. The dramatic loss of this subset from the peripheral blood in the NIgG group during peak viremia may be a consequence of apoptotic loss, consistent with increased CD95 expression, differentiation into other B cell phenotypes, and/or migration into other compartments. Interestingly, MZ B cells have been previously reported as being highly sensitive to HIV/SIV infection [19, 20, 35, 36], including a significant loss of splenic and lymph node MZ at 8 weeks p.i. of rhesus with SHIV_SF162P4_ [37]. Our findings that the IgD+CD5+CD21+ population is (i) exceptionally and rapidly responsive to SHIV infection, including significant loss from peripheral blood as soon as 3 days p.i., (ii) is preserved by SHIVIG treatment, (iii) shares phenotypic similarity with MZ-like B cells and (iv) is strongly associated with the kinetics of HIV Env-specific plasma antibody indicates that this population should be further evaluated as it may either directly or indirectly impact the induction and kinetics of HIV Env-specific antibody development.

## Conclusion

During the last several years, we and others have accumulated evidence highlighting the importance of early immunopathological events that occur during early HIV/SIV/SHIV infection: direct association between uncontrolled viral replication and memory T-cell loss (GALT) and B-cell dysregulation (polyfunctional activation and apoptosis) [17, 18, 23, 38–41]. In this study, we have investigated passively transferred polyclonal Abs with neutralizing activity (SHIVIG) and report their control of acute viral replication and mitigation of B cell dysregulation that consequently induced significantly greater and stronger endogenous Ab responses. We identified a specific B-cell population (MZ-like) that is highly and particularly affected during acute SHIV infection, and we identified a direct association with the preservation of this B cell subset and the production of endogenous Env-specific binding antibodies. Furthermore, we found that passive transfer of SHIVIG protected this particular MZ-like B cell subset and induced a higher Env-specific antibody response. The ability of MZ B cells to respond rapidly to pathogens, contribute to T- independent and T-dependent IgM antibody development, and serve as a major precursor to gut IgA-producing plasma cells [42–47], could be beneficial in the early stages of HIV infection. Our results indicate further evaluation of MZ B cells including their dissection for Env-specificity, and their selective targeting by HIV vaccination strategies should be evaluated. The precise identity of specific Abs responsible for these biological responses is not known. It is likely that the functional activity of SHIVIG is NAb, but we cannot rule out the contribution of other Abs in the mixture that can mediate ADCC, ADCP, or other activities. More work is needed to understand how each of these functions improves B cell health.

## Supporting information

S1 File Data. Contains study data.
(XLSX)
